# Structural alterations in the seminiferous tubules of rats treated with immunosuppressor tacrolimus

**DOI:** 10.1186/1477-7827-7-19

**Published:** 2009-02-25

**Authors:** Breno H Caneguim, Paulo S Cerri, Luís C Spolidório, Sandra M Miraglia, Estela Sasso-Cerri

**Affiliations:** 1 Department of Morphology and Genetics, Federal University of São Paulo (UNIFESP), São Paulo, Brazil; 2 Department of Morphology, Laboratory of Histology and Embryology, Dental School – São Paulo State University (UNESP), Araraquara, Brazil; 3 Department of Physiology and Pathology, Dental School – São Paulo State University (UNESP), Araraquara, Brazil

## Abstract

**Background:**

Tacrolimus (FK-506) is an immunosuppressant that binds to a specific immunophilin, resulting in the suppression of the cellular immune response during transplant rejection. Except for some alterations in the spermatozoa, testicular morphological alterations have not been described in rats treated with tacrolimus. In the present study, we purpose to evaluate if the treatment with tacrolimus at long term of follow-up interferes in the integrity of the seminiferous tubules.

**Methods:**

Rats aging 42-day-old received daily subcutaneous injections of 1 mg/kg/day of tacrolimus during 30 (T-30) and 60 (T-60) days; the rats from control groups (C-30 and C-60) received saline solution. The left testes were fixed in 4% formaldehyde and embedded in glycol methacrylate for morphological and morphometric analyses while right testes were fixed in Bouin's liquid and embedded in paraffin for detection of cell death by the TUNEL method. The epithelial and total tubular areas as well as the stages of the seminiferous epithelium and the number of spermatocytes, spermatids and Sertoli cells (SC) per tubule were obtained.

**Results:**

In the treated groups, seminiferous tubules irregularly outlined showed disarranged cellular layers and loss of germ cells probably due to cell death, which was revealed by TUNEL method. In addition to germ cells, structural alterations in the SC and folding of the peritubular tissue were usually observed. The morphometric results revealed significant decrease in the number of SC, spermatocytes, spermatids and significant reduction in the epithelial and total tubular areas.

**Conclusion:**

Tacrolimus induces significant histopathological disorders in the seminiferous tubules, resulting in spermatogenic damage and reduction in the number of Sertoli cells. A careful evaluation of the peritubular components will be necessary to clarify if these alterations are related to the effect of FK-506 on the peritubular tissue.

## Background

The immunosuppressive therapy in organ transplant recipients has included the use of calcineurin inhibitors such as tacrolimus (FK-506) and cyclosporine [1]. Although the mechanism of action of tacrolimus is similar to that of cyclosporine, tacrolimus is 10 to 100 times more potent and has been referred as useful therapeutic option for the transplant recipients. Tacrolimus is a macrolide immunosuppressant isolated from fermentation broth of *Streptomyces tsukubaensis *[2]. This drug binds to a specific immunophilin, called FKBP12, forming a complex in the lymphocytes that inhibits calcineurin, a Ca^2+^- and calmodulin-dependent serine/threonine phosphatase. Calcineurin plays a critical role in interleukin (IL)-2 promoter induction after T-cell activation [3]. Thus, the tacrolimus-FKBP12 complex prevents dephosphorylation of the cytoplasmic subunit of nuclear factor of activated T-cell (NF-ATc) by calcineurin, resulting in the suppression of IL-2 transcription and inhibition of the cellular immune response during transplant rejection [1]. High levels of tacrolimus in the blood are observed 2 hours after oral administration [3,4]. This drug (98%) circulates in the blood, bound to plasma proteins, such as albumin, lipoproteins and globulins [4,5] and accumulates in several tissues such as lung, spleen, kidney, heart, pancreas, muscle and liver [1,3].

Some adverse effects associated to tacrolimus treatment have been related in the treated patients, such as nephrotoxicity, neurotoxicity, *Diabetes mellitus*, gastrointestinal disturbances and hypertension [1,6,7]. The inhibition of calcineurin leads to decrease in the intracellular transcription of insulin by the pancreatic cells. Thus, hyperglicemia and post-transplant *Diabetes mellitus *(PTDM) are the most common metabolic diseases caused by calcineurin inhibitors such as FK-506 [1,8]. The nephrotoxicity has been associated to vasoconstriction, reduction in the glomerular filtration [9] and histopathological alterations, such as hyaline arteriolopathy, striped interstitial fibrosis and tubular atrophy [10-12].

Studies focusing the effects of calcineurin inhibitor immunosuppressants, mainly tacrolimus, on the reproductive system are scarce in the literature. Some testicular effects have been demonstrated in rats treated with the other calcineurin inhibitor immunosuppressant – cyclosporine. Among the effects, reduction in the testicular weight [13], decrease in the diameters of the seminiferous tubules [13-16], decrease in the number of spermatocytes at stage VII [17] and desquamation of round spermatids [16,17] have been reported. Additionally, testicular ultrastructural analyses revealed the presence of damaged spermatids, as well as disconnection of the head from the tail of spermatozoa and abnormal development of flagella [16]. In contrast to cyclosporine, testicular alterations have not been found in tacrolimus-treated rats for 2 weeks [18,19]. Moreover, the plasma levels of testosterone [18,19] and LH [18] were normal after tacrolimus treatment. However, the number and motility of spermatozoa reduced and the number of live fetus and the embryonic implantation index reduced after mating of the treated male rats with untreated female rats [19]. Since these authors have observed degenerative germ cells in the lumen of epididymis, it has been suggested that the quantitative and functional alterations in the spermatozoa resulted from the effect of tacrolimus in the epididymis rather than the seminiferous epithelium. According to the transplanted organ, the period of therapy in the patients receiving tacrolimus-based immunosuppression varies from 1 to 5 years of follow-up. In the present study, we purpose to evaluate the structural integrity of the seminiferous tubules in rats treated with tacrolimus at long term (30 and 60 days) of follow-up.

## Methods

### Animals and treatment

Twenty Holtzman male rats (*Rattus norvegicus albinus*) aging 42 day-old (weighing around 160 g) were maintained in plastic cages under 12 h light/12 h dark cycle at controlled temperature (23 ± 2°C), with water and food provided *ad libitum*. The animal care and the experimental procedures were conducted following the national law on animal use. This protocol was approved by the Ethical Committee for Animal Research of São Paulo Federal University, Brazil (UNIFESP/EPM).

The animals were distributed into four groups: two control groups (C-30 and C-60) and two tacrolimus (Prograf^® ^– Janssen Cilag, São José dos Campos, SP, Brazil) groups (T-30 and T-60). The rats from T-30 and T-60 were weighed weekly and received daily subcutaneous injections of 1 mg/kg/day of tacrolimus [20] during 30 and 60 days, respectively. This dosage provides plasma peak and through levels of tacrolimus of approximately 11.2 ng/ml [21-23] and has been reported to be immunosuppressive and clinically relevant, resulting in a consistent response [20].

The animals from control groups received subcutaneous injections of saline solution during the same periods.

### Histological procedures

The rats were anaesthetized with 80 mg/Kg of body weight of Ketamine (Francotar^®^; Virbac do Brazil Ind. e Com. Ltda., São Paulo, SP, Brazil). The testes were removed, weighed and the rats were killed by an overdose of Ketamine. The right testes were fixed in Bouin's liquid, embedded in paraffin and the sections were submitted to TUNEL method for detection of cell death. The left testes were fixed in 4% formaldehyde (prepared from paraformaldehyde), at pH 7.4 with 0.1 M sodium phosphate, embedded in glycol methacrylate (Historesin-Embedding Kit, Jung, Germany) and the sections were stained with Gill's hematoxylin and eosin – HE [24] for morphometric analyses.

### Morphometric analyses

Seminiferous tubules cross sections were randomly chosen in three non-serial sections per animal, totalizing 100 tubules/animal. Taking into account that the epithelial area can be altered without to affect, necessarily, the total tubular area, these two areas were evaluated. In each tubule, the epithelial area and the total tubular area were measured by using a 25-point integrating eyepiece attached to a light binocular microscope (Carl Zeiss, Germany). The number of points upon the seminiferous epithelium and upon the whole seminiferous tubule (epithelium and lumen) was counted at ×250. Thus, the epithelial and total tubular areas were obtained by multiplying the number of points by the area of each point [25].

Based on the method of classification previously described [26,27], the seminiferous tubules were classified into four categories according to the total area: a) reduced (measuring 19 × 10^3 ^μm^2 ^– 51 × 10^3 ^μm^2^), b) small (measuring 51 × 10^3 ^μm^2 ^– 83 × 10^3 ^μm^2^), c) medium (83 × 10^3 ^μm^2 ^– 115 × 10^3 ^μm^2^) and d) large (measuring 115 × 10^3 ^μm^2 ^– 147 × 10^3 ^μm^2^). The frequency of tubules according to these categories was obtained in all groups.

The identification of the stages of the seminiferous epithelium in the rat has been greatly improved when the testes are embedded in glycol methacrylate [28]. Thus, during the morphometric analysis, the stages I to V; VI to VIII; IX; X; XI to XIV [29] were identified and the frequency of tubules according to these stages was obtained. In the treated animals, only seminiferous tubules whose stages could be identified were computed.

The number of germ cells and Sertoli cells were quantified in cross sections of seminiferous tubules. The number of Sertoli cells per tubule was computed in 30 tubules/animal [30] using a light binocular microscope at ×500 (Carl Zeiss, Germany). Only Sertoli cells exhibiting typical morphological nuclear features and evident nucleolus were quantified. The number of spermatocytes and spermatids (round and elongate) was quantified in 10 tubules/animal [17]. Since the number of germ cells in tubular sections at stages I-VIII is different from those at stages IX-XIV, five tubules at stage I-VIII and five tubules at stage IX-XIV per animal were counted.

### TUNEL method

For detection of DNA breaks in the dying germ cells, we used the TUNEL (**T**erminal deoxynucleotidyl transferase-mediated d**U**TP **N**ick-**E**nd **L**abeling) method. The Apop Tag Peroxidase kit (Chemicon International, Chemicula, CA, USA) for in situ apoptosis detection was used [31]. The sections were treated with 3% hydrogen peroxide to block endogenous peroxidase and DNA end-labeling was performed by the incubation of sections in TdT enzyme. Subsequently, the sections were incubated with anti-digoxigenin-peroxidase antibodies; the reaction was revealed by 0.06% 3.3-diaminobenzidine (DAB) and the sections were counterstained with Carazzi's hematoxylin. Sections of mammary gland provided by the manufacturer of the kit were used as positive controls for the TUNEL method. Testicular sections, used as negative controls, were incubated in a TdT enzyme-free solution.

### Statistical analysis

Statistical analysis of morphometric data was performed using the software SigmaStat 3.2. According to the distribution of data, the differences between groups were analyzed by the t-Student test or the Mann-Whitney test. The significance level accepted was p ≤ 0.05.

For assessment of time-dependent action of tacrolimus, the total tubular area was analyzed by two-way ANOVA test.

## Results

### Body and testicular weight

According to table 1, the body weight of animals from T-30 and T-60 reduced significantly. A significant reduction in the absolute testicular weight was observed only in T-60 group.

**Table 1:** Body weight (BW) and absolute testicular weight (TW) of animals from control (C-30 and C-60) and tacrolimus (T-30 and T-60) groups

**Animals**	**BW (g)**	**TW (g)**
**C-30**	330 ± 34	1.66 ± 0.10
**T-30**	265 ± 20*	1.58 ± 0.15
**C-60**	423 ± 21	1.85 ± 0.10
**T-60**	310 ± 22*	1.65 ± 0.05*

*p < 0.05(statistically significant)

### Light microscopy

**Figure 1:**
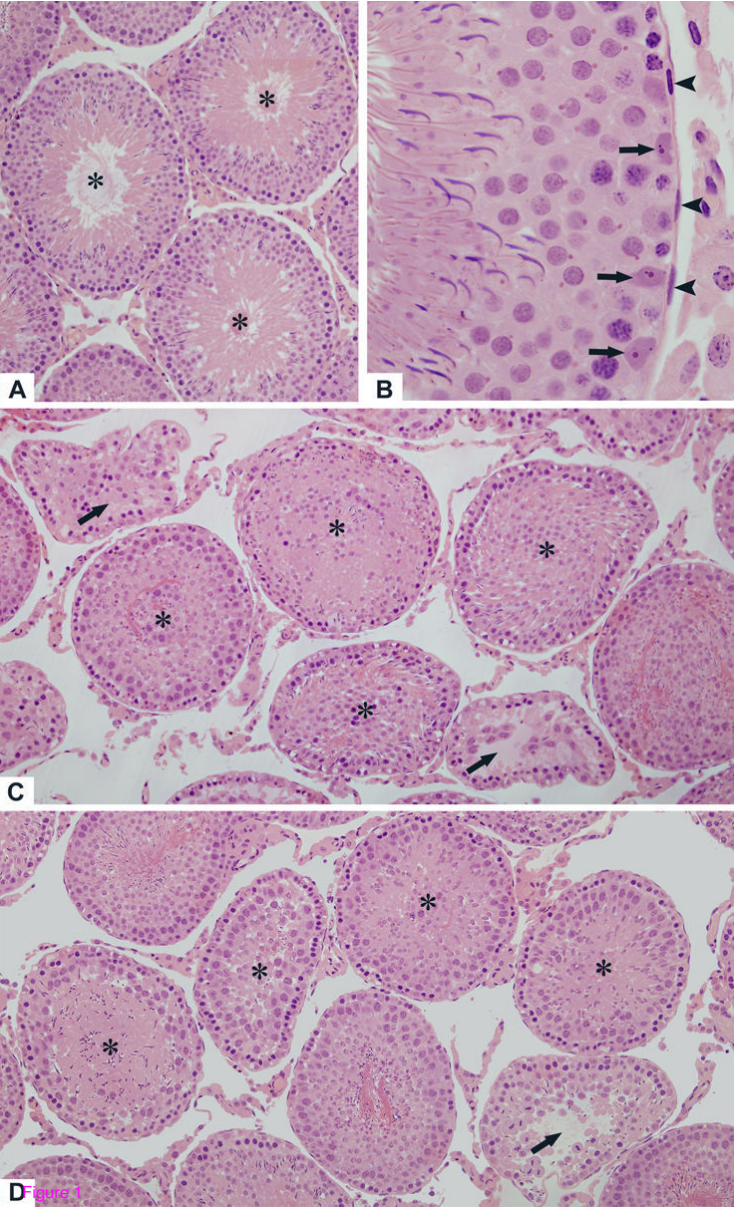
**Photomicrographs of seminiferous tubules of rats from C-30 (A), C-60 (B), T-30 (C) and T-60 (D) groups stained by H.E**. (**A **and **B**) In the seminiferous tubules with normal aspect, the germ cells are organized in concentric layers and the tubular lumen is empty (**A**, asterisks); (**B**) The Sertoli cell nuclei (arrows) are positioned adjacent to the well defined peritubular tissue in which peritubular cells are observed (arrowheads). In T-30 (**C**) and T-60 (**D**), the altered seminiferous tubules show irregular shape, epithelial disorganization and detached germ cells filling the tubular lumen (asterisks). In some atrophied seminiferous tubules, loss of germ cells is observed (arrows). Figs. 1A, 1C and 1D: ×110; Figs. 1B: ×330.

**Figure 2:**
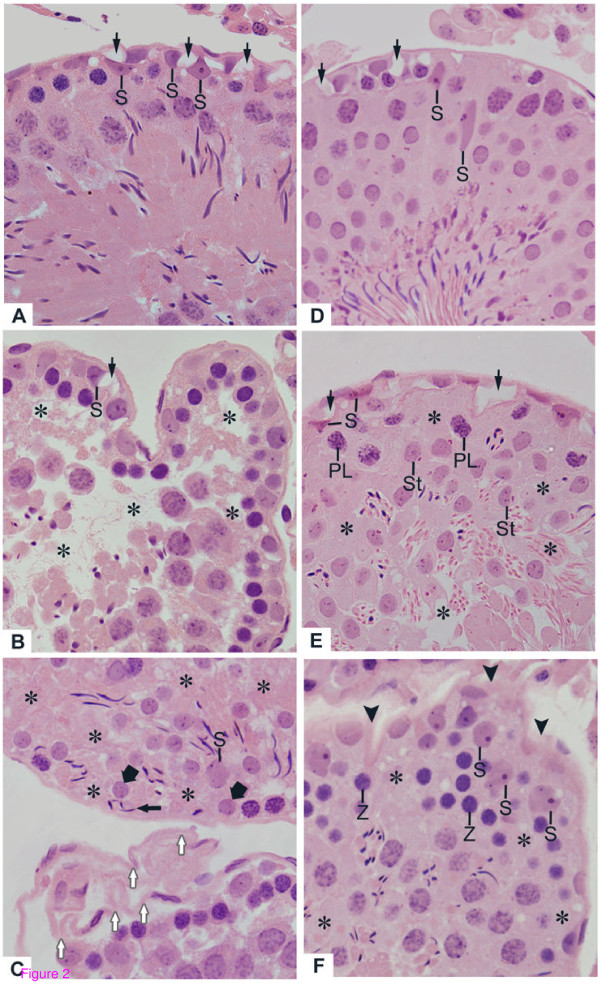
**Photomicrographs of seminiferous tubules of rats from T-30 (A, B and C) and T-60 (D, E and F) groups stained by H.E**. (**A **and** B**) Slightly (**A**) and severely (**B**) damaged tubules show vacuolar spaces (arrows) adjacent to Sertoli cell nuclei with irregular shape (S). In **B**, intraepithelial spaces (asterisks) due to lack of spermatocytes and spermatids were observed. (**C**) Lack of germ cells (asterisks) is noted in the basal and adluminal portions. Round (thick arrows) and elongate (thin arrow) spermatids are abnormally positioned in the basal compartment. Note a single Sertoli cell showing dislocated nucleus (S). In the other tubule, the peritubular tissue is intensely folded (white arrows). (**D**) Slightly altered seminiferous tubule shows vacuolar spaces (arrows) adjacent to Sertoli cell nuclei. Some irregular Sertoli cell nuclei (S) are displaced from their original site. (**E **and **F**) The damaged tubules show lack of germ cells (asterisks) in the layers of round spermatids (St, in **E**), pre-leptotene (PL, in **E**) and zygotene (Z, in **F**) spermatocytes. In **E**, vacuolar spaces (arrows) adjacent to irregular and strongly stained Sertoli cell nuclei (S) are observed. In **F**, the peritubular tissue is irregularly outlined (arrowheads). Adjacent to this altered tissue, the Sertoli cell nuclei are irregular and displaced from their original site (S). Figs. 2A-2F: ×330.

In the testicular sections of rats from C-30 and C-60, the seminiferous tubules with normal shape showed germ cells organized in concentric layers and the tubular lumen was usually empty. Typical triangle/ovoid Sertoli cell nuclei exhibiting evident nucleolus were observed in the basal compartment adjacent to peritubular tissue. The peritubular tissue surface was rectilinear and showed several nuclei of peritubular cells (Figs. 1A and 1B). In the testicular sections of tacrolimus-treated rats (T-30 and T-60), seminiferous tubules irregularly outlined showing disarranged epithelial layers and lumen filled with detached germ cells were observed; lack of germ cells were evident in the atrophied tubules (Figs. 1C and 1D). In the altered tubules, the Sertoli cell nuclei exhibited irregular shape and strongly stained chromatin. Adjacent to these altered nuclei, vacuolar spaces were often observed (Figs. 2A, 2B, 2D and 2E). Moreover, nuclei of Sertoli cell displaced from their normal position to the adluminal compartment were found in tubules at different stages (Figs. 2C, 2D and 2F). Disorganized round and elongate spermatids irregularly positioned in the basal compartment and intraepithelial spaces indicating loss of germ cells were found (Figs. 2B, 2C, 2E and 2F). Surrounding the altered epithelium, the peritubular tissue was irregularly outlined and, sometimes, intensely folded (Figs. 2C and 2F).

**Figure 3:**
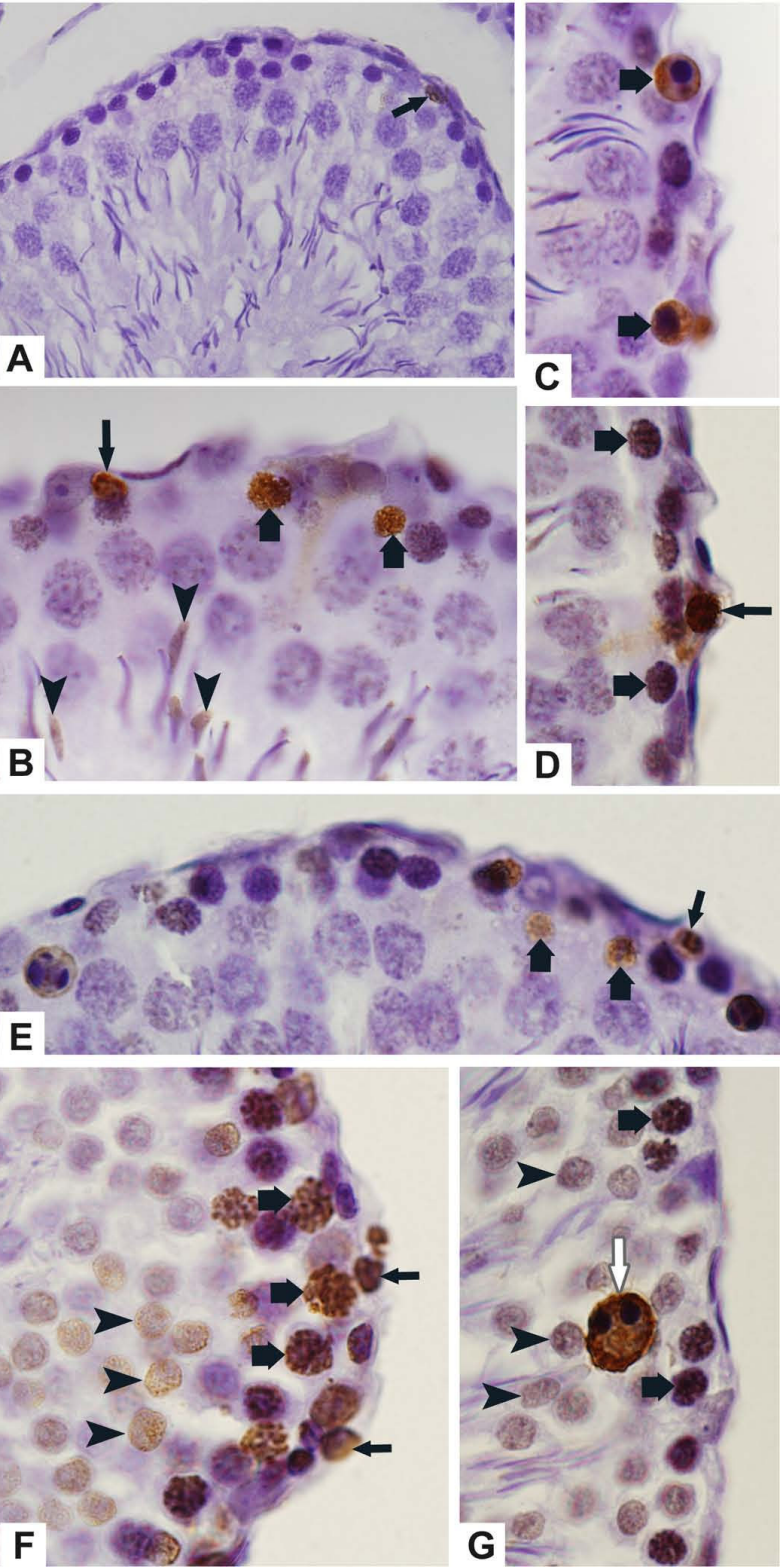
**Photomicrographs of seminiferous tubules of rats from C-30 (A), T-30 (B-D) and T-60 (E-G) submitted to the TUNEL-method**. (**A**) TUNEL-positive germ cell is observed in the epithelium (arrow). (**B-D**) Primary spermatocytes (thick arrows), spermatogonia (thin arrows) and elongate spermatids (arrowheads) are labeled by TUNEL method. (**E-G**) Spermatogonia (thin arrows), primary spermatocytes in different stages (thick arrows) and round spermatids (arrowheads) are TUNEL-positive. A giant multinucleated cell derivative from round spermatids (white arrow) is also positive. Fig. 3A: ×260; Figs. 3B-3G: ×710.

The testicular sections of rats from control groups submitted to TUNEL method revealed scarce TUNEL-positive germ cells (Fig. 3A). Otherwise, TUNEL-labeling was usually observed in spermatogonia, leptotene to pachytene spermatocytes as well as in round and elongate spermatids of the tacrolimus-treated rats (Figs. 3B–3G). TUNEL-positive giant multinucleated cells derivative from round spermatids were also found (Fig. 3G).

The sections of mammary gland, used as positive control, showed numerous TUNEL-positive cells while none positivity was observed in the testicular sections used as negative control (data not illustrated).

### Morphometric results

**Figure 4:**
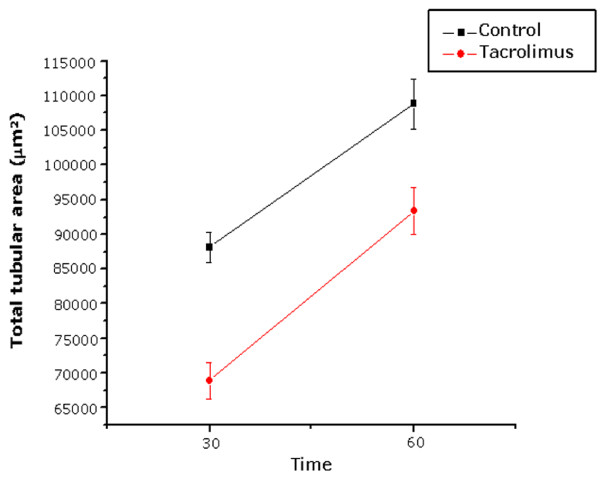
**Effect of FK-506 upon total tubular area (μm^2^) of the seminiferous tubules according to the time of treatment**.

A significant reduction in the tubular and epithelial areas was observed in the testes of rats treated with tacrolimus during 30 and 60 days, in comparison to the animals from C-30 and C-60, respectively (Table 2). The total tubular area increased significantly under normal conditions, i.e. from C-30 (72 days-old) to C-60 (102 days-old). However, the analysis of the effect of tacrolimus on the seminiferous tubules according to the periods of treatment revealed that the increase in the tubular area from T-30 to T-60 was proportional to the increase of the tubular area of control group (C-30 to C-60). There was no statistical difference regarding the increase of the tubular area, from 30 to 60 days, between control and tacrolimus groups (Fig. 4).

**Table 2:** Total tubular area (TA), seminiferous epithelium area (EA) and number of Sertoli cells (SC), spermatocytes (Sp) and spermatids (St) per tubule of animals from control (C-30 and C-60) and tacrolimus (T-30 and T-60) groups

**Animals**	**TA (μm^**2**^)**	**EA (μm^**2**^)**	**SC/tubule**	**Sp/tubule**	**St/tubule**
**C-30**	88,167 ± 4,885	81,587 ± 4,766	12.36 ± 0.49	118.6 ± 9.9	228.9 ± 13.7
**T-30**	68,889 ± 5,739*	64,051 ± 5,712*	10.22 ± 0.38*	70.7 ± 8.9*	94.8 ± 20.0*
**C-60**	108,812 ± 8,044	101,580 ± 7,035	13.00 ± 0.48	102.1 ± 11.1	207.2 ± 20.2
**T-60**	93,376 ± 7,455*	87,478 ± 7,160*	9.70 ± 0.14*	72.8 ± 9.0*	141.6 ± 7.1*

*p < 0.05(statistically significant)

**Figure 5:**
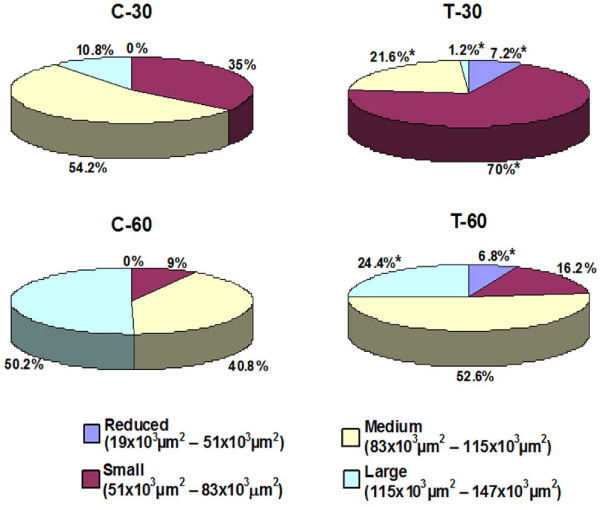
**Frequency (%) of seminiferous tubules according to the total tubular area (μm^2^) of animals from control (C-30 and C-60) and tacrolimus (T-30 and T-60) groups**. *p < 0.05 (statistically significant).

According to figure 5, the tubular area, in the control groups (C-30 and C-60), varied from 51 × 10^3 ^μm^2 ^to 147 × 10^3 ^μm^2^. After the analysis of the frequency of tubules according to the area, the results revealed in C-30 and C-60, respectively: a) 35% and 9% of small tubules (51 × 10^3 ^μm^2 ^to 83 × 10^3 ^μm^2^), b) 54% and 40% of median tubules (83 × 10^3 ^μm^2 ^to 115 × 10^3 ^μm^2^) and c) 10% and 50% of large tubules (measuring over 115 × 10^3 ^μm^2^). However, in the treated animals (T-30 and T-60), only 58% and 74% of the total tubules, respectively, showed a similar frequency pattern to those of the control groups. In T-30, the frequency of median and large tubules decreased significantly (60% and 89%, respectively), resulting in the significant increase (100%) in the frequency of small tubules. In T-60, the frequency of large tubules reduced significantly (51%), resulting in the increase in the frequency of both median (29%) and small (80%) tubules. It is important to note that in T-30 and T-60, respectively, the treatment resulted in the frequency of 6.8% and 7.2% of tubules measuring less than 51 × 10^3 ^μm^2 ^(reduced tubules), which were not found in the control groups.

According to table 2, the number of Sertoli cells/tubule reduced significantly (18.6% and 25.4%) in the testes of T-30 and T-60 groups, respectively. There was a significant reduction in the number of spermatocytes and spermatids per tubule in both tacrolimus groups. However, the reduction was more accentuated in T-30 (40% and 58%) than in T-60 (28% and 31%).

Although the frequency of tubules at stages VI-VIII decreased 42% in T-60, the differences regarding the frequency of seminiferous tubules according to the stages were not statistically significant between control and tacrolimus groups (table 3).

**Table 3:** Frequency (%) of seminiferous tubules according to the stages of cycle of seminiferous epithelium of animals from control (C-30 and C-60) and tacrolimus (T-30 and T-60) groups

**Stages**	**C-30**	**T-30**	**C-60**	**T-60**
**I to V**	44.6 ± 4.7	39.4 ± 4.6	36.2 ± 9.4	47.0 ± 6.2
**VI to VIII**	22.2 ± 4.4	21.0 ± 9.5	25.0 ± 13.0	14.4 ± 5.7
**IX**	5.8 ± 1.1	7.5 ± 2.4	6.2 ± 2.6	6.5 ± 4.2
**X**	2.4 ± 0.9	5.4 ± 4.1	3.8 ± 1.5	3.1 ± 1.6
**XI to XIV**	25.0 ± 3.0	23.0 ± 18.5	28.8 ± 4.4	29.0 ± 9.4

## Discussion

Similar to previous findings [19,32], the results revealed a significant reduction in the body weight of rats from treated groups. Regarding the effect of tacrolimus on the spermatogenesis, the treatment of rats with 1 or 3 mg/kg/day of tacrolimus (FK-506) for 2 weeks leads to sperm counts and motility decrease. However, histopathological findings were not observed in the testes and due to the presence of degenerative germ cells in the epididymis, it has been concluded that spermatozoa were affected in the epididymis, but not in the testes [19]. Spermatozoa are originated from the spermatogenic process in the seminiferous epithelium which is the target tissue for the action of many drugs. However, data regarding the effect of FK-506 on the seminiferous tubules have not been found in the literature. In contrast to the findings of the previous study [19], significant alterations were observed in the seminiferous epithelium of the rats treated with 1 mg/kg/day of tacrolimus during 30 and 60 days. These differences could be due to the long periods of treatment evaluated in this study, in contrast to the short period [19].

The treatment with the immunosuppressor FK-506 induces alterations in the histoarchitecture of the seminiferous epithelium, resulting in germ cell layers disarrangement, abnormal positioning of disordered spermatids in the tubular basal compartment, detached germ cells filling the tubular lumen and significant decrease in the number of spermatocytes and spermatids. These alterations resulted in a significant reduction in the epithelial and total tubular areas after both periods of treatment indicating that the reduction in the testicular weight is actually due to a pathologic response to the treatment. The treatment with cyclosporine, a calcineurin inhibitory immunosuppressor, has caused similar testicular alterations [13-16]; among them, depletion of seminiferous epithelium due to loss of germ cells and decrease in the tubular diameter has been described [16,17]. In the present study, although the mean value of tubular area reduced significantly in the treated groups, we verified that some tubules were intensely altered, while others were apparently normal or slightly altered. It is important to consider that, normally, the diameters of seminiferous tubules in cross sections are variable due to the fact that the different segments of tubules contain cells of germinal lineage in different stages of the seminiferous cycle [33]. For this reason, the classification of tubules in sizes according to area was necessary to compare control and treated groups. Based on this analysis, it was possible to conclude that, in T-30, the reduction in the tubular area was due to the significant reduction in the frequency of large and median tubules, resulting in a high frequency of small tubules. Similarly, the increased frequency of small and median tubules was probably due to the reduction in the frequency of large tubules in T-60. Based on this analysis, we could verify that 42% and 26% of seminiferous tubules in T-30 and T-60, respectively, were affected by tacrolimus, resulting in the increased frequency of small tubules in these groups. Moreover, some tubules were severely affected by tacrolimus, resulting in the frequency of 7% of reduced tubules (measuring less than 51 × 10^3 ^μm^2^) which were inexistent in the control groups.

It has been demonstrated that cellular adhesive junctions are important for the maintenance of tubular shape and volume [34]. Reduction in the tubular size associated to detachment and loss of germ cells by cell death have been observed in the testis of rats treated with different drugs [31,35-37]. Decrease in the number of spermatocytes [17] and desquamation of round spermatids [16,17] have been reported in the seminiferous tubules of rats treated with the immunosuppressant cyclosporine. In the present study, a significant reduction in the number of spermatocytes and spermatids was observed in the treated animals (T-30 and T-60). Moreover, the TUNEL reaction was positive in several cells of the germinal lineage. Taken together, these findings indicate that the immunosuppressive treatment with tacrolimus results in the loss of germ cells by cell death and, then, in the reduction of the tubular area.

The nutritional and structural support of germ cells is maintained by Sertoli cells; after Sertoli cell structural injury, the Sertoli cell-germ cell physical interaction is disrupted [38] and programmed cell death is induced in the detached germ cells [39]. In the present study, Sertoli cell nuclei showed abnormal shape and, sometimes, were displaced from their original site. Sertoli cell nuclei can be found positioned away from the basement membrane in normal tubules at stage VIII [28]; however, in the present study, the Sertoli cell nuclei were dislocated from the basal to adluminal compartment in tubules at different stages. In addition, the number of Sertoli cells reduced significantly in the treated groups and a probable occurrence of Sertoli cell death should be further investigated. Thus, it is possible that germ cell loss is due, at least in part, to Sertoli cell damage; this is reinforced by the fact that abnormal Sertoli cell nuclei were also found in the tubules slightly altered by tacrolimus. Moreover, the presence of disordered spermatids in the tubular basal compartment suggests phagocytosis by Sertoli cells and, then, failure of release; this abnormal condition can be caused by a primary change in the Sertoli cells [40]. Structural alterations and apoptosis in the Sertoli cells have been related to damage in the integrity of the peritubular tissue [30]. In the current study, the seminiferous tubules showed irregularly outlined peritubular tissue and, in some portions, this tissue was intensely folded. In part, these alterations could be resulted from loss of germ cells and tubular shrinkage. However, a possible interference of tacrolimus on the peritubular components and, subsequently, on the Sertoli and germ cells, should be considered and further investigated. This hypothesis is reinforced by the fact that the results revealed no susceptibility of the seminiferous epithelium to FK-506, according to the stage of the seminiferous cycle.

The analysis of the effect of tacrolimus on the testes according to the periods of treatment (30 and 60 days) leads to conclude that the effect of FK-506 was more accentuated in the first period of treatment (T-30). In T-30, the total tubular area reduced significantly and the number of small tubules increased 100%, in comparison to control group; however, after more 30 days of treatment (T-60), the frequency of these tubules did not change significantly and the increase in the tubular area was proportional to the increase in the tubular area of control group (from C-30 to C-60). Under normal conditions, the animals from C-30 (aging 72-day-old) contain around 11% of large tubules, while the animals from C-60 (aging 102-day-old) contain around 50% of large tubules, indicating that the seminiferous tubules develop and increase their size after 30 days. In contrast, only 24% of large tubules were found in T-60. This reduction in the frequency of large tubules was due to the accentuated effect of tacrolimus in the number of spermatocytes and spermatids, which decreased 40% and 58%, respectively, in T-30. Thus, the effect of tacrolimus on the integrity of the seminiferous epithelium was more accentuated in the beginning of the treatment (T-30), resulting in a failure in the tubular development following the treatment (T-60).

It has been demonstrated that calcineurin/NF-AT plays a role in the function of pancreatic β-cells, including in the cellular proliferation and synthesis of insulin [41]. Thus, inhibition of calcineurin leads to decreased insulin transcription [42,43] and, then, to post-transplantation *Diabetes mellitus *– PTDM [44], the most known adverse effect caused by immunosuppressive treatment with tacrolimus [1,6,7]. The effect of diabetes on the male reproductive system has been related to sexual impotence [45] and male infertility [46]. Studies of rats induced to diabetes have demonstrated testicular alterations such as tubular atrophy due to loss of germ cells [45,47] and high frequency of apoptotic germ cells [46,48]. Moreover, ultrastructural alterations in the peritubular tissue of the seminiferous tubules of diabetic man have also been demonstrated [47]. Cases of PTDM have been reported in patients after 1 month of the therapy with tacrolimus [49]. Moreover, decreased tubular diameter, hyalinized tubular walls and epithelial alterations have also been observed in rats induced to diabetic condition for only 5 days [50]. In a previous study, high glycemic levels have been detected in T-30 (data not published) and in T-60 [51]. The abnormalities in the glucose metabolism are normalized after tacrolimus therapy for 180 and 240 days [51]. Therefore, future studies are necessary to establish a possible relationship between the tubular alterations, observed in the treated rats, and a possible diabetogenic effect, induced by FK-506.

## Conclusion

Preventive caution must be taken during tacrolimus therapy in male transplant recipients since this immunosuppressor induces histopathological disorders in the seminiferous tubules, resulting in a significant decrease in the number of germ cells. The spermatogenic damage can be related to morphological and quantitative alterations in the Sertoli cells. Future ultrastructural analyses of the peritubular tissue are necessary to confirm if the epithelial alterations are resulted from a possible effect of tacrolimus on the peritubular components.

## Competing interests

The authors declare that they have no competing interests.

## Authors' contributions

ESC coordinated the study. LCS carried out the treatments of animals. BHC, ESC and PSC collected and carried out the histological processing. BHC carried out the histological staining, TUNEL method and morphological and morphometric analyses. BHC, PSC, ESC and SMM examined and selected the images. All authors participated in the design, writing, read and approved the final manuscript.
